# Down-regulation of long non-coding RNA HOTAIR promotes angiogenesis via regulating miR-126/SCEL pathways in burn wound healing

**DOI:** 10.1038/s41419-020-2247-0

**Published:** 2020-01-23

**Authors:** Bimei Jiang, Yuting Tang, Hao Wang, Cheng Chen, Wenchang Yu, Hui Sun, Mengting Duan, Xiaofang Lin, Pengfei Liang

**Affiliations:** 1 Department of Burns and Plastic Surgery, Xiangya Hospital, Central South University, Changsha, Hunan China; 2 Department of Pathophysiology, Xiangya School of Medicine, Central South University, Changsha, Hunan China; 3 Sepsis Translational Medicine Key Laboratory of Hunan Province, Xiangya School of Medicine, Central South University, Changsha, Hunan China

**Keywords:** Collective cell migration, Trauma

## Abstract

miR-126, an endothelial-specific microRNA, is associated to vascular integrity and angiogenesis. It is well established that angiogenesis plays a critical role in burn wound healing. However, there was a lack of understanding of the mechanism by which miR-126 regulates angiogenesis during burn wound healing. HOX transcript antisense intergenic RNA (HOTAIR) is a well-characterized long non-coding RNA (lncRNA) involved in cell proliferation, apoptosis, migration, and invasion of cancer cells. Sciellin (SCEL), a precursor to the cornified envelope of human keratinocytes, has been shown to inhibit migration and invasion capabilities of colorectal cancer cells. In this study, a cohort of 20 burn wound tissues and paired adjacent normal tissues were collected. LncRNA and messenger RNA expression profiles were screened by microarray analysis in five pairs of samples with mostly increased miR-126 levels. miR-126 was highly expressed in burn wound tissues and human umbilical vein endothelial cells (HUVECs) exposed to heat stress, whereas HOTAIR and SCEL were down-regulated after thermal injury. Bioinformatic analysis, dual luciferase reporter assay, and quantitative real-time PCR were conducted to validate that HOTAIR and SCEL competitively bind to miR-126 to function as the competitive endogenous RNA. miR-126 promoted endothelial cell proliferation, migration, and angiogenesis, but suppressed apoptosis, while HOTAIR and SCEL exerted opposite effects in HUVECs. The biological functions were determined by MTT (3-(4,5-dimethylthiazol-2-yl)-2,5-diphenyltetrazolium bromide) assay, Annexin-V-FITC/PI (propidium iodide/fluorescein isothiocyanate) staining, transwell migration, and tube formation assays. Collectively, our study revealed that HOTAIR/miR-126/SCEL axis contributes to burn wound healing through mediating angiogenesis.

## Introduction

Burn injuries could be induced by thermal agents, as well as radiations or chemicals. Severe burn injuries induce complex pathologic effects on nearly every organ system, and lead to ~180,000 death annually worldwide^1^. Millions of burn patients suffer from both physical and psychological morbidities. The post-burn mortality has been decreased over the past few decades due to the improvement of resuscitation procedures, advancements in infection prevention, and burn wound healing management^2,3^. The complex wound healing process consists of four highly integrated and overlapping phases, including hemostasis, inflammation, proliferation, and tissue remodeling^4^. Angiogenesis is a critical activity of proliferative phase during wound healing. This includes a number of cellular events, such as endothelial cell proliferation and endothelial cell migration, as well as the formation and maturation of capillary-like tubules into new blood vessels^5^. To improve current burn wound healing management, it is important to gain a better understanding of the underlying mechanisms involved in angiogenesis.

Differentially expressed microRNAs (miRNAs) were identified in burned tissues, including the known oncogenic miRNA miR-126^6^. Emerging evidence suggest that miR-126, which is highly expressed in endothelial cells, plays important roles in angiogenesis and maintenance of vascular integrity^7,8^. miR-126 exhibits multiple properties under different conditions. For instance, miR-126 stimulates angiogenic signaling in fetal organisms by targeting vascular endothelial growth factor (VEGF) signaling suppressors Sprouty-related protein (SPRED1) and phosphoinositol-3 kinase regulatory subunit 2 (PIK3R2)^7,9^. In vessel injury or hypoxia, overexpression of miR-126 induces angiogenesis, while it maintains vascular homeostasis by inhibiting angiogenesis in adult organisms^10,11^. Sciellin (SCEL), which is originally characterized as a precursor to the cornified envelope of human keratinocytes^12^, has been shown to inhibit migration and invasion capabilities of colorectal cancer cells by regulating Wnt signaling^13^. In this study, we predicted SCEL as a direct target of miR-126 using bioinformatics analysis. Microarray analysis revealed that SCEL was down-regulated in burned tissues, indicating that miR-126/SCEL might play an important role in burn wound healing.

Long non-coding RNAs (lncRNAs) are a type of non-coding RNAs that exceed 200 nucleotides in length. It is well established that lncRNAs exhibit a variety of biological functions by transcriptional and post-transcriptional regulation of target genes^14^. Salmena et al.^15^ proposed the competitive endogenous RNA (ceRNA) hypothesis, illustrating that lncRNA and messenger RNA (mRNA) competitively bind to miRNA. LncRNA acts as a ceRNA through harboring miRNA, thus modulating miRNA target gene expression^16^. Angiogenesis depends on precise gene regulation; however, the role of lncRNA in angiogenesis remains poorly understood^17^. As a well-characterized lncRNA, HOX transcript antisense intergenic RNA (HOTAIR) is involved in cell proliferation, apoptosis, migration, and invasion of cancer cells^18^. Recently, it has been reported that HOTAIR suppresses angiogenesis of human placenta by inhibiting VEGFA^19^. Nevertheless, whether HOTAIR function as a sponge of miR-126 to regulate angiogenesis during burn wound healing process needs to be elucidated.

In this study, we hypothesized that HOTAIR/miR-126/SCEL axis might play crucial roles in regulating endothelial function based on bioinformatics and microarray analysis. Our results revealed a novel molecular mechanism of burn wound healing, indicating that HOTAIR/miR-126/SCEL might be a potential therapeutic target of burn patients.

## Materials and methods

### Collection of human normal and burned tissues

Normal uninjured and burn wound tissues were obtained from 20 patients with deep partial thickness burn in Xiangya Hospital. The samples were collected during tangential excision of eschar and large sheets of split thickness autograft with preservation of dermis on the fourth day after burn. Consents from all patients were obtained. Approval of this study was obtained from the Research Ethics Board at Xiangya Hospital (No. 201403302).

### Cell culture

Human umbilical vein endothelial cells (HUVECs) and HEK293T cells were obtained from Cell Bank of Type Culture Collection, Chinese Academy of Science (Shanghai, China). Cells were cultured in RPMI1640 medium containing 10% fetal bovine serum (FBS, Gibco, Thermo Fisher Scientific, Waltham, MA, USA), 100 μg penicillin, and 100 U/ml streptomycin. Cultures were maintained at 37 °C in a humidified environment with 5% CO_2_.

### Heat treatment

HUVECs were seeded in culture dishes 48 h prior to the heat treatment. For the heat stress treatment, cells were harvested into a 15 ml centrifuge tube and immersed in a circulating water bath temperature regulated at 52 °C for 2.5 or 3 min. For control group, cells were put in a water bath at 37 °C for 2.5 or 3 min. Heat-treated cells were re-seeded in culture dishes and further incubated at 37 °C. Cells were then harvested at 6 h after heating.

### Cell transfection

HUVECs were seeded in culture dishes 24 h prior to the transfection. For enhanced or decreased expression of miR-126, miRNA mimics control (mimics NC), miR-126 mimics, miRNA inhibitor control (inhibitor NC), or miR-126 inhibitor were purchased from GenePharma (Suzhou, Jiangsu, China). miRNAs were transfected into HUVECs using Lipofectamine 2000 reagent (Thermo Fisher Scientific) according to the manufacturer’s instructions. At 24 h, cells were harvested for subsequent analysis.

### Lentivirus/Adenovirus packaging and transduction

The lentivirus vectors (Lv-SCEL) and adenovirus vector (Ad-HOTAIR) constructed by UCBio (Changsha, Hunan, China) were used for stable overexpression or knockdown of SCEL and HOTAIR in HUVECs, respectively. HUVECs were transduced with SCEL overexpression/shRNA (short hairpin RNA) lentivirus or HOTAIR overexpression/shRNA adenovirus according to the manufacturer’s instructions, and selected with puromycin (Sigma-Aldrich, St. Louis, MO, USA) for 2–3 weeks to obtain stable cell lines.

### Microarray analysis

Five pairs of samples were prepared for lncRNA microarray analysis using Human lncRNA microarray v.4.0 (Arraystar, Rockville, MD, USA). The slides were incubated for 17 h at 65 °C in an Agilent hybridization oven and scanned by the Agilent Scanner G2505B, Agilent Feature Extraction software (version 11.0.1.1) was utilized to analyze acquired array images. Quantile normalization and subsequent data processing were conducted using GeneSpring GX v.12.1 software package (Agilent Technologies, Santa Clara, CA, USA). Differentially expressed lncRNAs and mRNAs between the two groups were identified through paired *t* test *P* <0.05 and a fold change >2.0. All microarray work was performed by Kangcheng Bio-Tec (Shanghai, China).

### Quantitative PCR (qRT-PCR)

Total RNA was isolated from HUVECs or tissues using Trizol reagent (Thermo Fisher Scientific) and reverse transcribed with Advantage RT-for-PCR Kit (TaKaRa, Dalian, China). RT products were used as templates for quantitative real-time PCR (qRT-PCR) with specific primers. The primers were designed using NCBI online tool Primer-BLAST (www.ncbi.nlm.nih.gov/tools/primer-blast). The following primers were used in this study: HOTAIR, forward 5′-GCAGTGGGGAACTCTGACTC-3′ and reverse 5′-AACTCTGGGCTCCCTCTCTC-3′; SCEL, forward 5′-TGGTCTCTGGCTAGAGTTAGCAATAA-3′ and reverse 5′-CCACCACTCACAGCCAACAT-3′; miR-126, forward 5′-GGAATGTAAGGAAGTGTG-3′ and reverse 5′-GAGCAGGCTGGAGAA-3′; GAPDH, forward 5′-CCAGGTGGTCTCCTCTGA-3′ and reverse 5′-GCTGTAGCCAAATCGTTGT-3′. The mRNA level of the target gene was analyzed by SYBR Premix EX Taq (TaKaRa) (*n* = 3, each in triplicates) and ABI PRISM 7900 Real-Time PCR System (Applied Biosystems, Foster City, CA, USA). Glyceraldehyde 3-phosphate dehydrogenase (GAPDH) was used as an internal control for lncRNA and mRNA. U6 was used as an internal control for miRNA. The specificity of the fluorescent signal was verified by both melting-curve and gel electrophoresis. The expression level of the target gene was determined using 2^−ΔΔCT^ method.

### Western blotting

Protein lysates from HUVECs or tissues were prepared in IP lysis buffer supplemented with protease and phosphatase inhibitor cocktail (Pierce, Thermo Fisher Scientific). Total protein concentration was determined by BCA Protein Assay Kit (Pierce, Thermo Fisher Scientific). Equal amount of protein lysates were resolved by sodium dodecyl sulfate-polyacrylamide gel electrophoresis and transferred onto PVDF membrane (Millipore, Bedford, MA, USA) for western blotting analysis. The membrane was blocked with 5% non-fat milk for 1 h at room temperature, followed by incubation with primary antibodies at 4 °C overnight. Primary antibodies used in this study included SCEL (1:2000; Abcam, Cambridge, UK), Col1a2 (1:2000; Abcam), MMP9 (1:2000; ABclonal, Cambridge, MA, USA), and VEGFA (1:2000; Abclonal). The membrane was then incubated with secondary antibody (1:10,000) for 1 h at room temperature. ECL western blotting detection reagents (Beyotime, Haimen, China) were used for protein detection. The X-ray films were scanned and bands were analyzed.

### Cell proliferation assay

Cell viability was determined by 3-(4,5-dimethylthiazol-2-yl)-2,5-diphenyltetrazolium bromide (MTT) assay (Sigma-Aldrich). HUVECs were seeded in 96-well plates at a density of 1 × 10^4^/plate. Cells were then transfected/transduced with miRNA or lentivirus/adenovirus. At different time points, a total of 20 μl MTT was added into each well and incubated for 4 h at 37 °C. The culture medium was then removed, and MTT formazan crystals were resolved in 150 μl dimethyl sulfoxide. Absorbance was measured at a wavelength of 490 nm by the use of microplate reader (Molecular Device, Menlo Park, CA, USA).

### Transwell migration assay

Transwell migration assay was performed using transwell chambers (Corning, NY, USA) with 6.5 mm diameter polycarbonate filters. At 24 h post transfection, cells were serum starved for 6 h. HUVECs were then seeded in the top chambers in triplicate. The bottom chambers were filled with RPMI1640 containing 10% FBS. Cells were allowed to migrate for 12 h. The remaining cells in the top chamber were carefully removed with cotton swabs. The migrated cells were fixed with 4% paraformaldehyde (PFA), stained with 0.1% crystal violet for 15 min, and counted under a Zeiss inverted microscope.

### Cell apoptosis assay

Cell apoptosis assay was performed using Annexin-V-FITC/PI (propidium iodide/fluorescein isothiocyanate) Apoptosis Assay Kit (Thermo Fisher Scientific) according to the manufacturer’s instruction. In brief, cells were resuspended in binding buffer. Cells were then incubated with 5 μl Annexin-V-FITC reagent and 5 μl PI in the dark for 15 min. The samples were analyzed by flow cytometry with BD FACSCalibur (BD Biosciences, San Jose, CA, USA).

### Endothelial cell tube formation assay

Tube formation assay was performed as previously described^20^. In brief, HUVECs transfected with miRNA or transduced with shRNA for 48 h were harvested and resuspended in 3 × 10^4^ cells per plate. One hundred microliters of cells were gently added to the Matrigel (BD Biosciences)-coated 96-well plates and incubated for 4 h at 37 °C. The capillary-like structures were acquired using Zeiss inverted microscope.

### Dual luciferase reporter assay

The wild-type (WT) or mutant of HOTAIR were constructed into pmirGLO vector, namely WT HOTAIR or MUT HOTAIR. The WT or mutant 3′-untranslated region (3′-UTR) of SCEL was constructed into pmirGLO vector, namely WT SCEL or MUT SCEL. The construct was co-transfected with miR-NC or miR-126 mimics into HEK293T cells using Lipofectamine 2000 transfection reagent (Thermo Fisher Scientific). The dual luciferase reporter assay was performed 36 h post transfection using Dual Luciferase Assay System (Promega, Madison, WI, USA). The luciferase activity of each sample was normalized to the Renilla luciferase activity. All experiments were performed in triplicate.

### Statistical analysis

All experiments were performed at least three times. Data are expressed as the means ± SD. Statistically significant differences were analyzed by Wilcoxon’s test. In selected experiments, a paired samples Wilcoxon’s test was used for paired comparisons. Statistical analysis was performed using the SPSS13.0 (SPSS Inc., Chicago, IL, USA). *P* < 0.05 was considered statistically significant.

## Results

### Analysis of miR-126 expression in denatured dermis

**Fig. 1:**
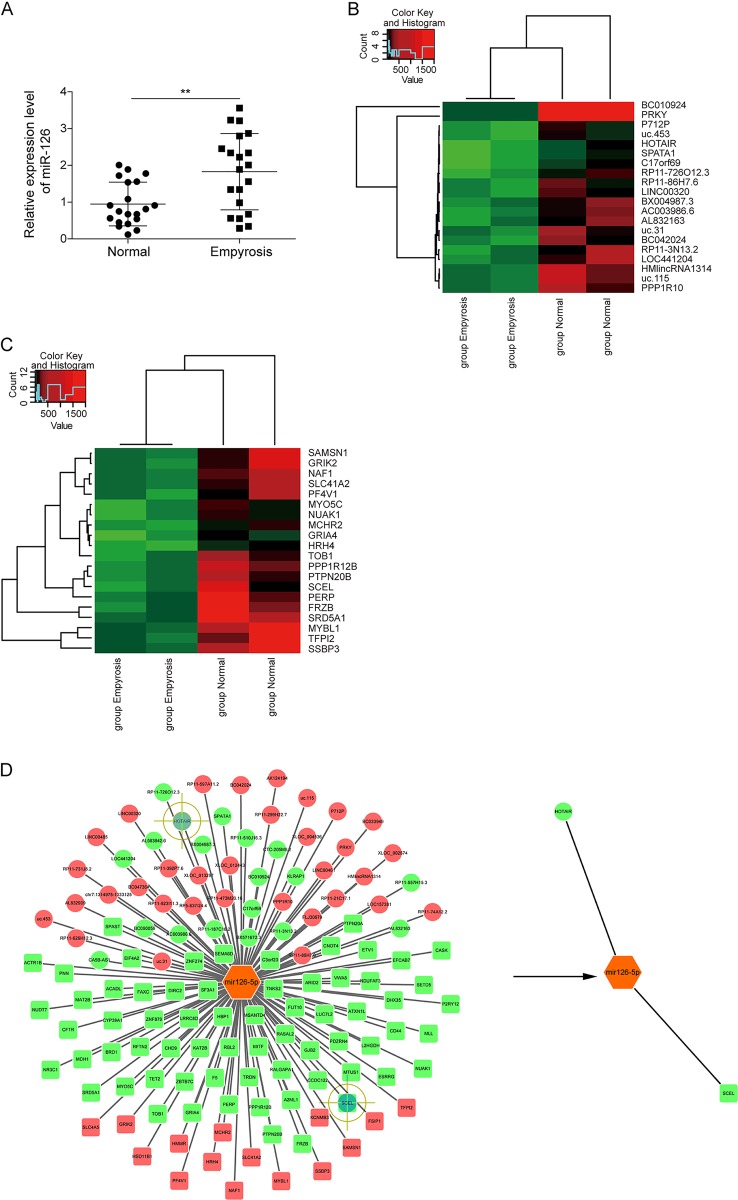
Profiles of differentially expressed genes in burn wound tissue compared to controls. **a** miR-126 levels in burn wound tissues and paired adjacent normal tissues were determined by qRT-PCR. U6 served as an internal control. **P < 0.01. **b** Top20 differentially expressed lncRNAs and **c** mRNAs in five pairs of samples with mostly increased miR-126 levels were subjected to hierarchical clustering. Red color indicated high relative expression and green color indicated low relative expression. **d** Construction of the miR-126, lncRNAs, and mRNA co-expression network. Red and green color represent up- and down-regulation, respectively.

To determine the level of miR-126 in burn wound tissues, a cohort of 20 patients with deep partial thickness burn were included. The results of qRT-PCR showed that miR-126 was significantly up-regulated in denatured dermis compared with those in normal skin (Fig. 1a).

### Analysis of differentially expressed lncRNAs/mRNAs in miR-126 up-regulated denatured dermis

Five pairs of samples with mostly increased miR-126 levels were selected for lncRNA microarray analysis. As shown in Fig. 1b, differentially expressed lncRNAs were discriminated between normal and burn wound tissues from the lncRNA expression profile. The results revealed 33,100 differentially expressed lncRNAs between these two groups, consisting of 19,600 lncRNAs up-regulated >1-fold in burn wound tissues compared to normal tissues, and 13,500 down-regulated lncRNAs. The heatmap as illustrated in Fig. 1b was constructed to show 20 mostly changed lncRNAs (*P* <0.05 and fold change >2) from the microarray analysis. Among these 20 dysregulated lncRNAs, 10 lncRNAs were decreased after burn injury, whereas 10 lncRNAs were increased. Notably, a 4.45-fold decrease was found in HOTAIR expression in burn wound tissues as compared to that in paired controls (Fig. 1b). In addition, 23,800 differentially expressed mRNAs were also analyzed. Compared to normal tissues, we found 9900 up-regulated and 13,900 down-regulated genes in burn wound tissues. As illustrated in Fig. 1c, 10 of top20 dysregulated genes were significantly down-regulated in burn wound tissues. The mRNA level of SCEL was decreased by 24.5-fold in burn wound tissues compared with paired controls. Furthermore, the gene co-expression networks were constructed to identify interactions among miR-126, mRNAs, and lncRNAs. A total lncRNAs and mRNAs containing relationships were selected to generate a network map (Fig. 1d). Taken together, the expression profile analysis of HOTAIR, miR-126, and SCEL in burn wound tissues indicate that HOTAIR might participate in the ceRNA network by sponging miR-126 to regulate SCEL expression.

### miR-126 promotes endothelial cell proliferation, migration, angiogenesis, and inhibits apoptosis

**Fig. 2:**
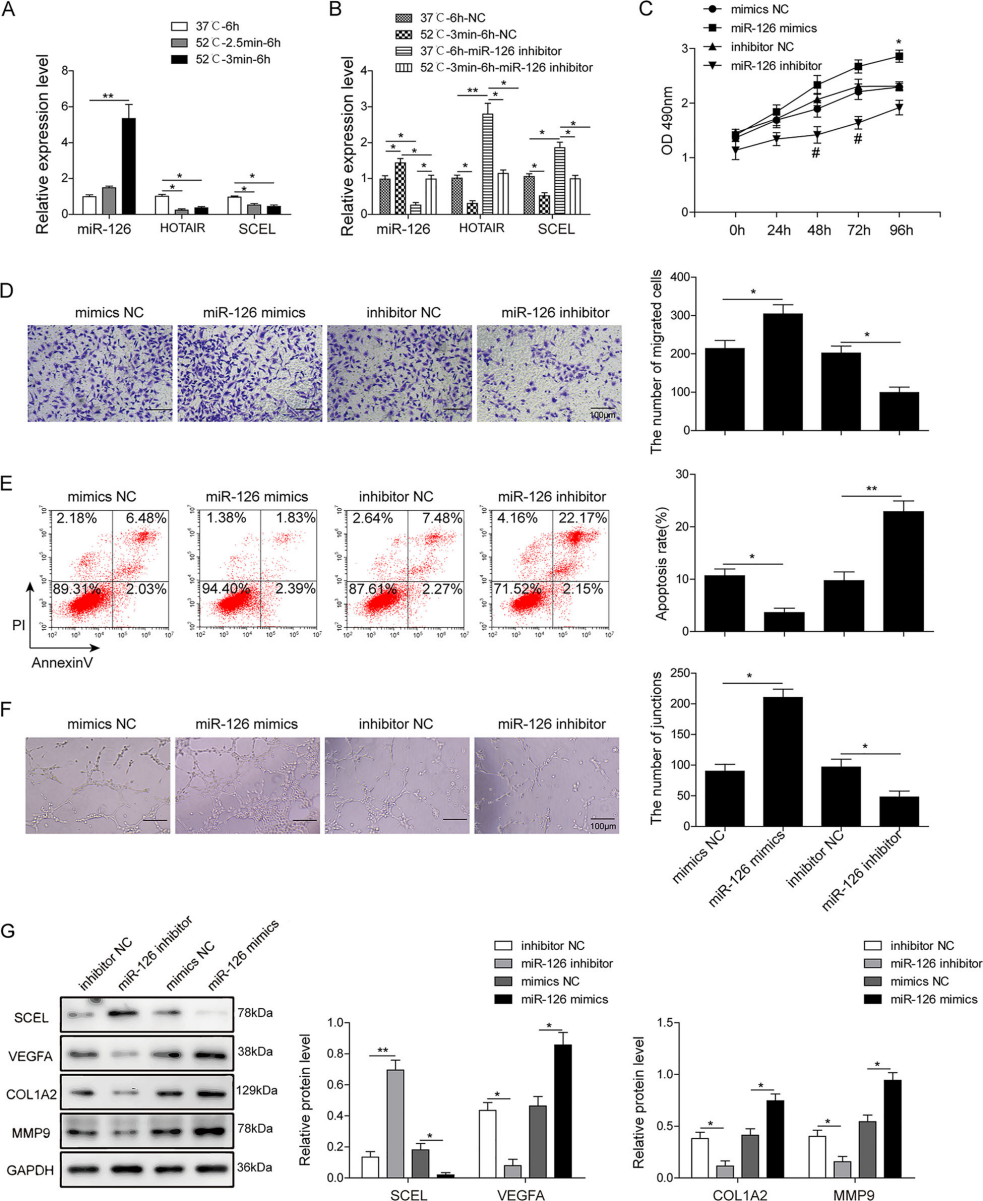
MiR-126 promotes endothelial cell proliferation, migration, angiogenesis, and inhibits apoptosis. **a** miR-126, HOTAIR, and SCEL levels in HUVECs exposed to heat stress were determined by qRT-PCR. U6 or GAPDH served as an internal control. **b** HUVECs were transfected with inhibitor NC or miR-126 inhibitor. miR-126, HOTAIR, and SCEL levels in HUVECs exposed to heat stress were determined by qRT-PCR. U6 or GAPDH served as an internal control. **c** Cell viability was monitored by the MTT assay. **d** Cell migration capacity was monitored by transwell migration assay. Data were representative images or were expressed as the mean ± SD of *n* *=* *3* experiments. **e** Cell apoptosis was determined by Annexin-V-FITC/PI staining followed by flow cytometry. **f** In vitro angiogenesis was quantified by tube formation assays. **g** Protein levels of SCEL, Col1a2, MMP9, and VEGFA were determined by western blotting. GAPDH served as loading control. Data were representative images or were expressed as the mean ± SD of *n* *=* 3 experiments. *P < 0.05 and **P < 0.01.

To investigate the role of miR-126 in burn wound healing in vitro, HUVECs were exposed to heat stress (52 °C) for either 2.5 or 3 min, and the cells were further incubated at 37 °C for 6 h. As shown in Fig. 2a, more significant changes in miR-126, HOTAIR, and SCEL levels were observed when HUVECs were exposed to heat stress for 3 min. This heat treatment condition was thus selected for subsequent experiments. To test the sponge interaction network between HOTAIR and SCEL in vitro, inhibitor (mimics) NC or miR-126 inhibitor (mimics) were transfected into HUVECs. Efficiency of miR-126 mimics or inhibitor transfection was determined by qRT-PCR (Fig. S1). Down-regulation of miR-126 caused an induction of HOTAIR and SCEL levels (Fig. 2b), indicating that miR-126 negatively regulates the expression of HOTAIR and SCEL. In addition, lack of miR-126 potentiated heat stress-induced reduction of HOTAIR and SCEL under the treatment of HUVECs at 52 °C for 3 min (Fig. 2b), suggesting that miR-126, HOTAIR, and SCEL might work in concert upon heat treatment. Gain-of-function and loss-of-function experiments were further performed. MTT and transwell migration assays showed that miR-126 mimics promoted cell proliferation and migration, whereas suppression of miR-126 inhibited cell growth and migration of HUVECs (Fig. 2c, d). Moreover, the apoptotic rate was markedly decreased by miR-126 mimics, but early and late apoptotic cells increased to 24.32% when the cells were transfected with miR-126 inhibitor (Fig. 2e). In vitro angiogenesis was quantified by tube formation assays. As shown in Fig. 2f, miR-126 mimics significantly enhanced the capability of HUVECs to form more complex and branched capillary-like structures. In contrast, miR-126 inhibitor group revealed shorter and broken tubes. Furthermore, western blotting confirmed that lack of miR-126 induced SCEL protein expression, but inhibited the levels of angiogenesis-related molecules VEGFA, Col1a2, and MMP9, whereas miR-126 mimics caused the opposite effects (Fig. 2g). Taken together, these findings indicate that down-regulation of miR-126 increased the expression of HOTAIR and SCEL, and miR-126 promotes endothelial cell proliferation, migration, angiogenesis, and inhibits apoptosis.

### HOTAIR and SCEL were down-regulated in denatured dermis

**Fig. 3:**
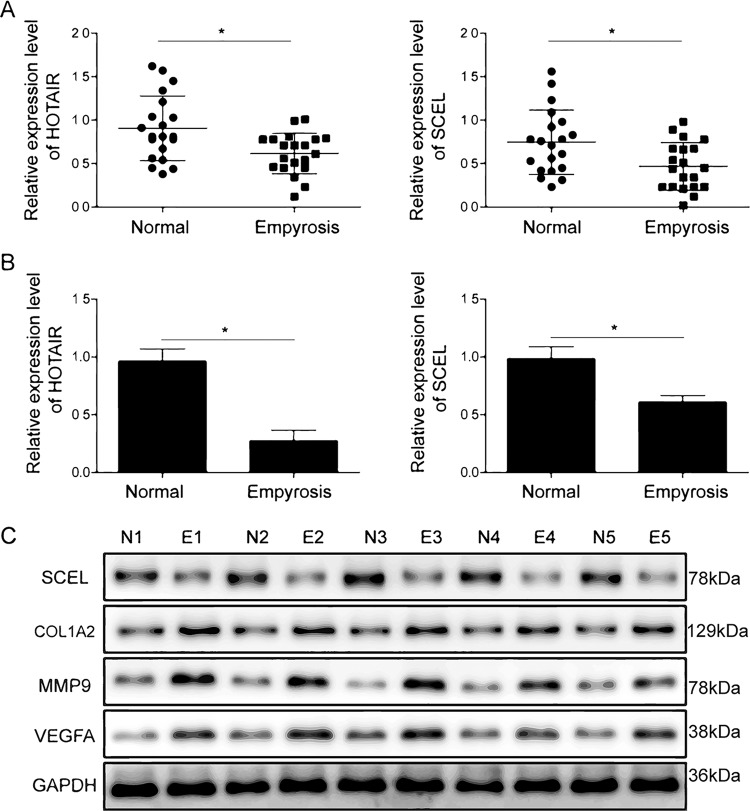
HOTAIR and SCEL was down-regulated after thermal injury. mRNA levels of HOTAIR and SCEL in clinical samples (**a**) and HUVECs (**b**) were determined by qRT-PCR. GAPDH served as an internal control. Each bar is a mean ± SD of *n* = 3 experiments. *P < 0.05. **c** Protein levels of SCEL, Col1a2, MMP9, and VEGFA in five pairs of samples with mostly increased miR-126 levels were determined by western blotting. GAPDH served as loading control. Data were representative images of *n* = 3 experiments.

To further verify the results of microarray analysis, qRT-PCR was performed to examine the mRNA levels of HOTAIR and SCEL. The results from qRT-PCR showed significant reduction in HOTAIR and SCEL levels consistent with microarray data (Fig. 3a, burn wound tissues vs. normal tissues). We further examined the HOTAIR and SCEL expression in HUVECs after heat stress. As shown in Fig. 3b, HOTAIR and SCEL expression levels showed significant decrease in post-heat stress. Similar to the mRNA expression, the reduction of SCEL was also observed by western blotting in five pairs of samples, which was subjected to microarray analysis (Fig. 3c). Moreover, burn injury dramatically up-regulated endothelial migration and angiogenesis-related molecules, including Col1a2, MMP9, and VEGFA (Fig. 3c). Taken together, these data suggest that thermal injury decreased the expression of HOTAIR and SCEL in both clinical samples and HUVECs. These might contribute to endothelial migration and angiogenesis.

### HOTAIR acts as miR-126 sponge

**Fig. 4:**
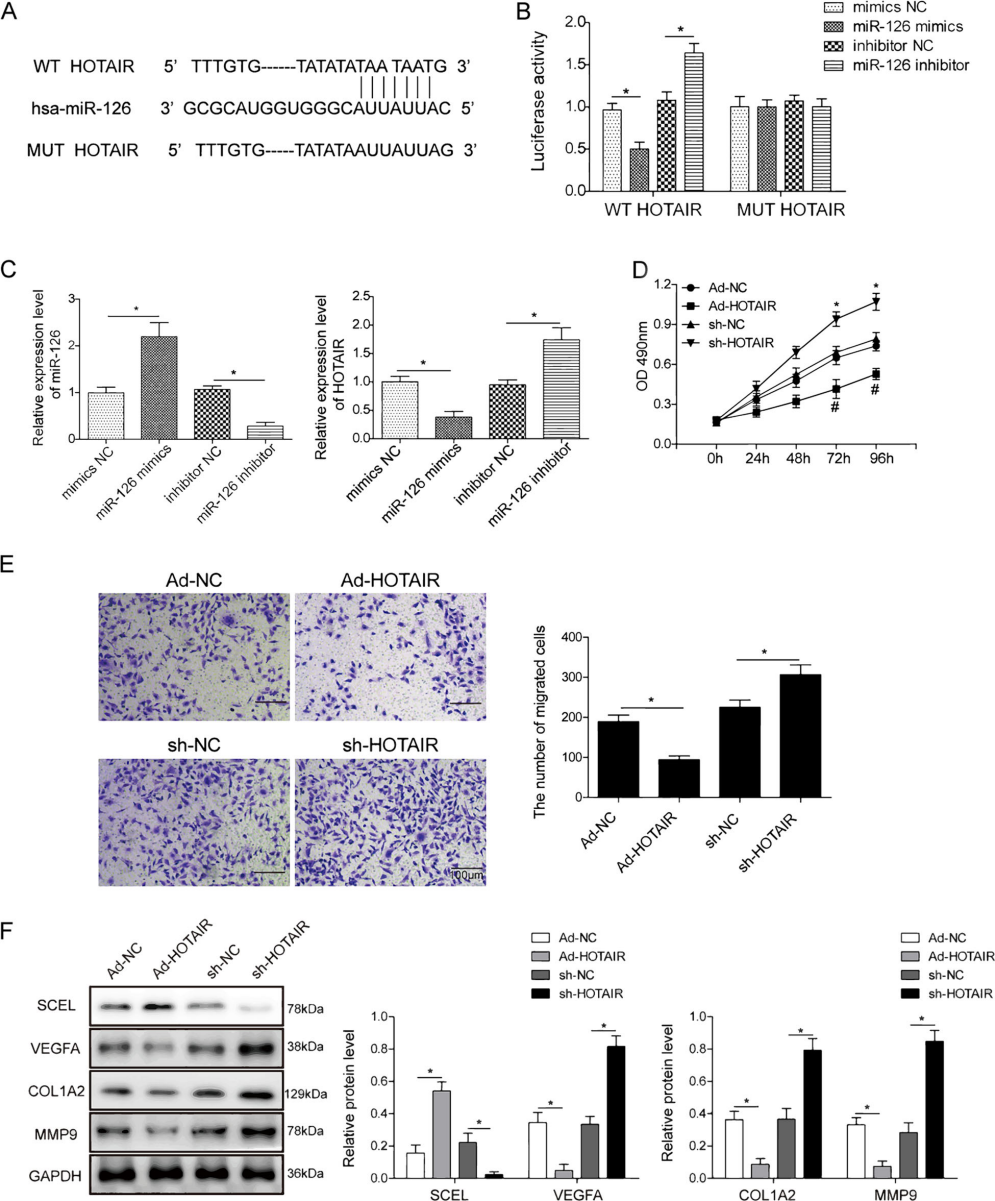
HOTAIR acts as miR-126 sponge and biological function of HOTAIR in HUVECs. **a** The predicted binding sites between miR-126 and HOTAIR. A mutation was generated in the HOTAIR sequence in the complementary site for miR-126 binding. **b** Co-transfection of WT/MUT HOTAIR and mimics NC/miR-126 mimics/inhibitor NC/miR-126 inhibitor in HEK293T cells. Firefly luciferase activity was examined by dual luciferase reporter assay. Renilla luciferase activity was used to normalize the activity of firefly luciferase. Data were representative images or were expressed as the mean ± SD of *n* = 3 experiments. **c** miR-126 and HOTAIR levels in HUVECs were determined by qRT-PCR. U6 or GAPDH served as an internal control. **d** Cell viability was monitored by the MTT assay. **e** Cell migration capacity was monitored by transwell migration assay. Data were representative images or were expressed as the mean ± SD of *n* = 3 experiments. **f** Protein levels of SCEL, Col1a2, MMP9, and VEGFA were determined by western blotting. GAPDH served as loading control. Data were representative images or were expressed as the mean ± SD of *n* = 3 experiments. *P < 0.05.

The results of microarray and qRT-PCR suggest that HOTAIR might be regulated by miR-126 through direct interaction. To verify this hypothesis, dual luciferase assay was performed to validate the direct interaction between HOTAIR and miR-126. As shown in Fig. 4a, the predicted miR-126 binding sites of HOTAIR, including both WT and mutants, were constructed into pmirGLO vector. Co-transfection of miR-126 mimics and WT HOTAIR led to a significant reduction of luciferase activity compared with mimics NC, whereas miR-126 inhibitor exerted opposite effect. In contrast, mutated HOTAIR (MUT HOTAIR) abolished the effect of either miR-126 mimics or inhibitor on luciferase activity (Fig. 4b), suggesting that miR-126 directly targets HOTAIR. We further examined HOTAIR level in HUVECs with different expression levels of miR-126. Overexpression of miR-126 mimics decreased HOTAIR, whereas down-regulated miR-126 increased HOTAIR significantly (Fig. 4c). Collectively, the results confirm the binding alignment between HOTAIR and miR-126, and miR-126 negatively regulated HOTAIR expression.

### Biological function of HOTAIR in HUVECs

In order to investigate whether HOTAIR plays a role in burn wound healing, we conducted experiments on monitoring the cell proliferation, migration, apoptosis, and tube formation abilities of HUVECs with different expression levels of HOTAIR. Adenoviral transduction efficiency was monitored via green fluorescent protein intensity, as well as qRT-PCR (Fig. S2). MTT assays showed the cell viability of HUVECs after adenoviral transduction. Overexpression of HOTAIR caused a significant decrease in cell proliferation, whereas knockdown of HOTAIR promoted cell growth (Fig. 4d). Transwell migration assay showed that HOTAIR intensively inhibited migration of HUVECs (Fig. 4e). The apoptotic rate increased more than 2-fold in HOTAIR-overexpressing cells, while it remarkably decreased in HOTAIR-knockdown cells (Fig. S3A). HOTAIR inhibited tube formation in HUVECs in which the number of capillary-like structures significantly decreased in HOTAIR-overexpressing cells, whereas lack of HOTAIR resulted in an extensive net of capillary-like structures (Fig. S3B). As expected, overexpression of HOTAIR increased SCEL protein level, but decreased the expression of angiogenesis-related molecules VEGFA, Col1a2, and MMP9 (Fig. 4f). Consistently, knockdown of HOTAIR presented the opposite results (Fig. 4f). In short, these results suggest that HOTAIR plays crucial roles in endothelial cell proliferation, migration, apoptosis, and angiogenesis. It is worth noting that the functions of HOTAIR and miR-126 in endothelial cells were opposite.

### MiR-126 mimics dissolves the anti-angiogenic effect mediated by HOTAIR

**Fig. 5:**
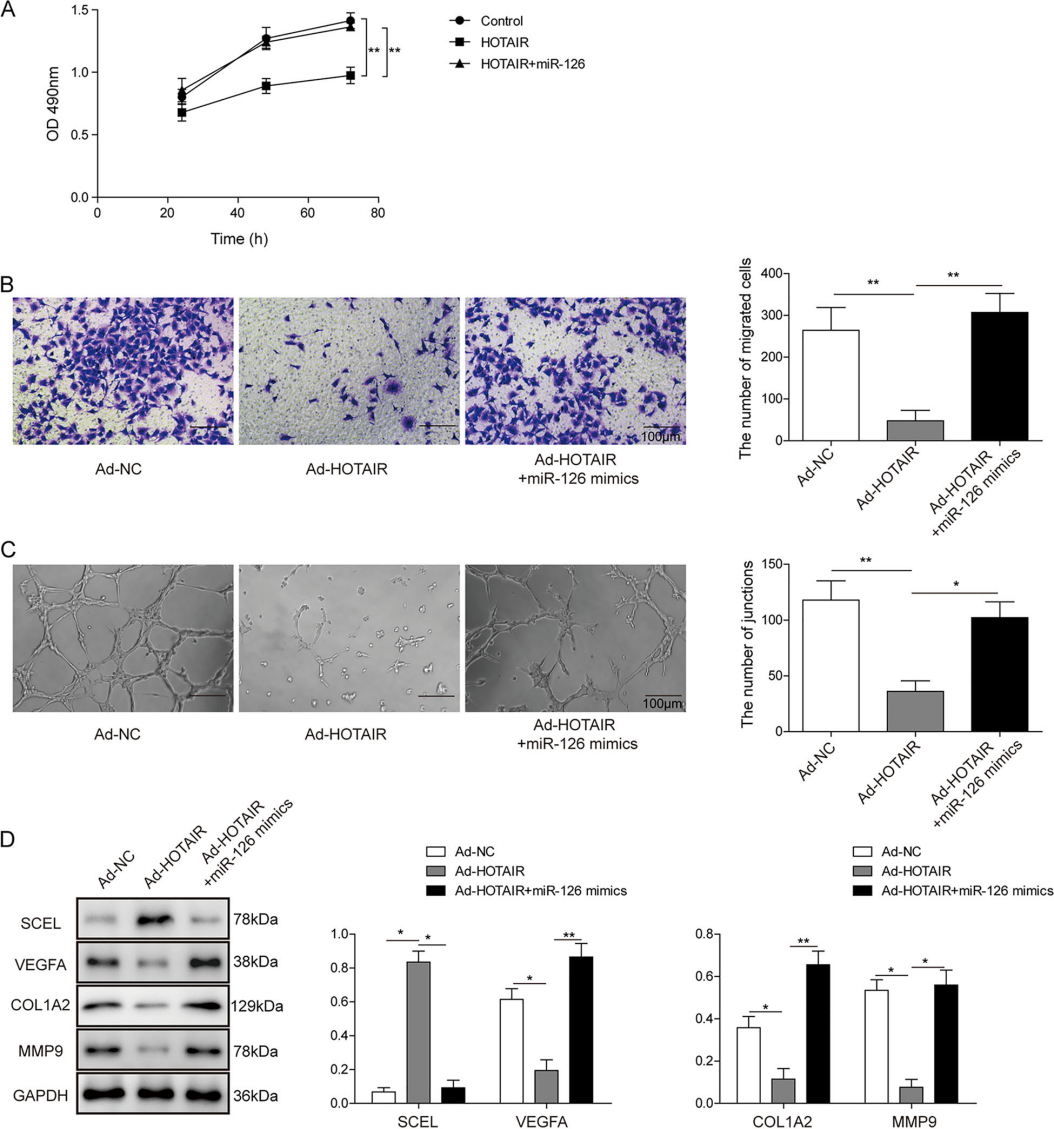
MiR-126 mimics dissolves the anti-angiogenic effect mediated by HOTAIR. **a** Cell viability was monitored by the MTT assay. **b** Cell migration capacity was monitored by transwell migration assay. Data were representative images or were expressed as the mean ± SD of *n* = 3 experiments. **c** In vitro angiogenesis was quantified by tube formation assays. **d** Protein levels of SCEL, Col1a2, MMP9, and VEGFA were determined by western blotting. GAPDH served as loading control. Data were representative images or were expressed as the mean ± SD of *n* = 3 experiments. *P < 0.05, **P < 0.01.

Our finding illustrated the binding alignment between HOTAIR and miR-126, and miR-126 negatively regulated HOTAIR expression raised the possibility that miR-126 levels may be the intermediate between HOTAIR and anti-angiogenic effect. To test this possibility, we investigated the effect of miR-126 mimics on anti-angiogenic effect mediated by HOTAIR. As shown in Fig. 5a, MTT assays showed that overexpression of HOTAIR caused a significant decrease in cell proliferation, whereas co-transfection of miR-126 mimics and WT HOTAIR led to a significant increase of cell proliferation. Then, transwell migration assay showed that HOTAIR intensively inhibited migration of HUVECs, and miR-126 mimics relieved the inhibition of migration mediated by HOTAIR in HUVECs (Fig. 5b). Also, miR-126 mimics relieved the inhibition of tube formation mediated by HOTAIR in HUVECs (Fig. 5c). In accordance with the previous experiment, western blotting revealed that miR-126 mimics attenuated the effects of HOTAIR on the protein levels of SCEL, and angiogenesis-related molecules VEGFA, Col1a2, and MMP9 (Fig. 5d). Taken together, these findings suggest that miR-126 plays a critical role in regulating HOTAIR-mediated anti-angiogenic effect.

### SCEL is a direct target of miR-126

**Fig. 6:**
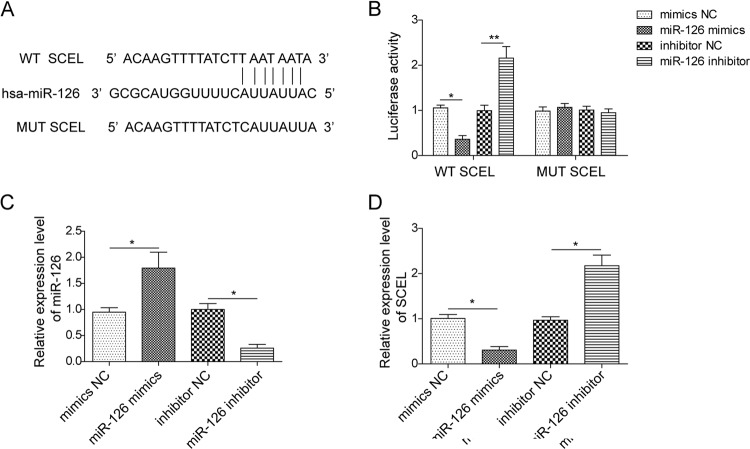
SCEL is a direct target of miR-126. **a** The predicted binding sites between miR-126 and SCEL 3′-UTR. A mutation was generated in 3′-UTR of SCEL in the complementary site for miR-126 binding. **b** Co-transfection of WT/MUT SCEL 3′-UTR and mimics NC/miR-126 mimics/inhibitor NC/miR-126 inhibitor in HEK293T cells. Firefly luciferase activity was examined by dual luciferase reporter assay. Renilla luciferase activity was used to normalize the activity of firefly luciferase. Data were representative images or were expressed as the mean ± SD of *n* = 3 experiments. **c**, **d** miR-126 and SCEL levels in HUVECs were determined by qRT-PCR. U6 or GAPDH served as an internal control. *P < 0.05, **P < 0.01.

We next sought to test if miR-126 can repress SCEL through direct 3′-UTR interactions. Bioinformatics analysis showed the complementary sequences between SCEL 3′-UTR and miR-126 (Fig. 6a). The WT or mutated 3′-UTR of SCEL was cloned into pmirGLO vector and dual luciferase reporter assays were performed in HEK293T cells. Co-transfection of miR-126 mimics and WT SCEL repressed luciferase activity significantly, while it failed to repress the luciferase activity in cells transfected with MUT SCEL. In contrast, MUT SCEL almost completely abolished miR-126 inhibitor-caused induction of luciferase activity when compared with corresponding controls (Fig. 6b). To determine whether miR-126 can repress endogenous SCEL, we firstly certified the transfection efficiency of miR-126 (Fig. 6c) and then examined SCEL expression in HUVECs by qRT-PCR (Fig. 6d). The data showed that miR-126 mimics repressed SCEL, whereas miR-126 inhibitor markedly increased SCEL mRNA level (Fig. 6d). These data reveal that miR-126 directly targets the 3′-UTR of SCEL, leading to repressed expression.

### SCEL inhibits endothelial cell proliferation, migration, angiogenesis, and promotes apoptosis

**Fig. 7:**
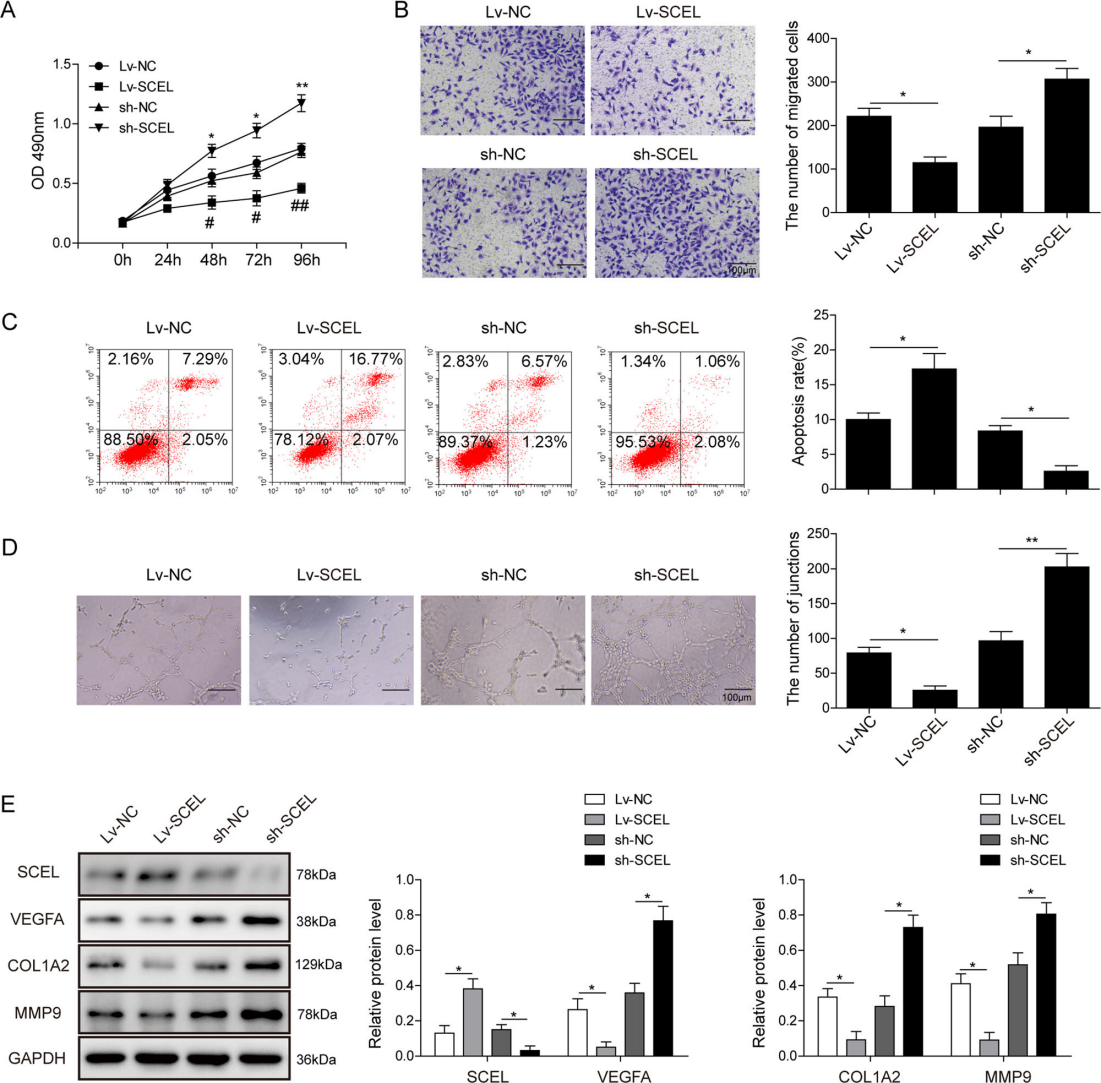
SCEL inhibits cell proliferation, migration, angiogenesis, and promotes apoptosis in HUVECs. **a** Cell viability was monitored by the MTT assay. **b** Cell migration capacity was monitored by the transwell migration assay. Data were representative images or were expressed as the mean ± SD of *n* = 3 experiments. **c** Cell apoptosis was determined by Annexin-V-FITC/PI staining followed by flow cytometry. **d** In vitro angiogenesis was quantified by tube formation assays. **e** Protein levels of Col1a2, MMP9, and VEGFA were determined by western blotting. GAPDH served as loading control. Data were representative images or were expressed as the mean ± SD of *n* = 3 experiments. *P < 0.05 and **P < 0.01.

To clarify the role of SCEL in HUVECs, we transduced the lentiviral SCEL expression vector or shRNA to HUVECs. Lentiviral transduction efficiency was monitored by qRT-PCR and Western blotting (Fig. S4). MTT assays revealed that SCEL overexpression decreased cell proliferation, whereas knockdown of SCEL restored this effect (Fig. 7a). Transwell migration assay showed that SCEL overexpression inhibited cell migration, but the increased migration ability was found in SCEL-knockdown cells (Fig. 7b). In addition, an increased rate of apoptosis was detected in SCEL-overexpressing cells, whereas lack of SCEL resulted in a significant inhibition of apoptosis (Fig. 7c). Overexpression of SCEL inhibited tube formation, while SCEL-knockdown HUVECs organized into capillary-like tubes interconnected in mesh-like structure (Fig. 7d). Consistently, overexpression of SCEL decreased the expression of VEGFA, Col1a2, and MMP9 (Fig. 7e). Taken together, SCEL inhibits cell proliferation, migration, tube formation, angiogenesis, and promotes apoptosis in HUVECs. SCEL and miR-126 exerted opposite effect in endothelial cells, further confirming the ceRNA hypothesis.

### SCEL reverses the regulation of miR-126 on endothelial cell proliferation, migration, and angiogenesis

**Fig. 8:**
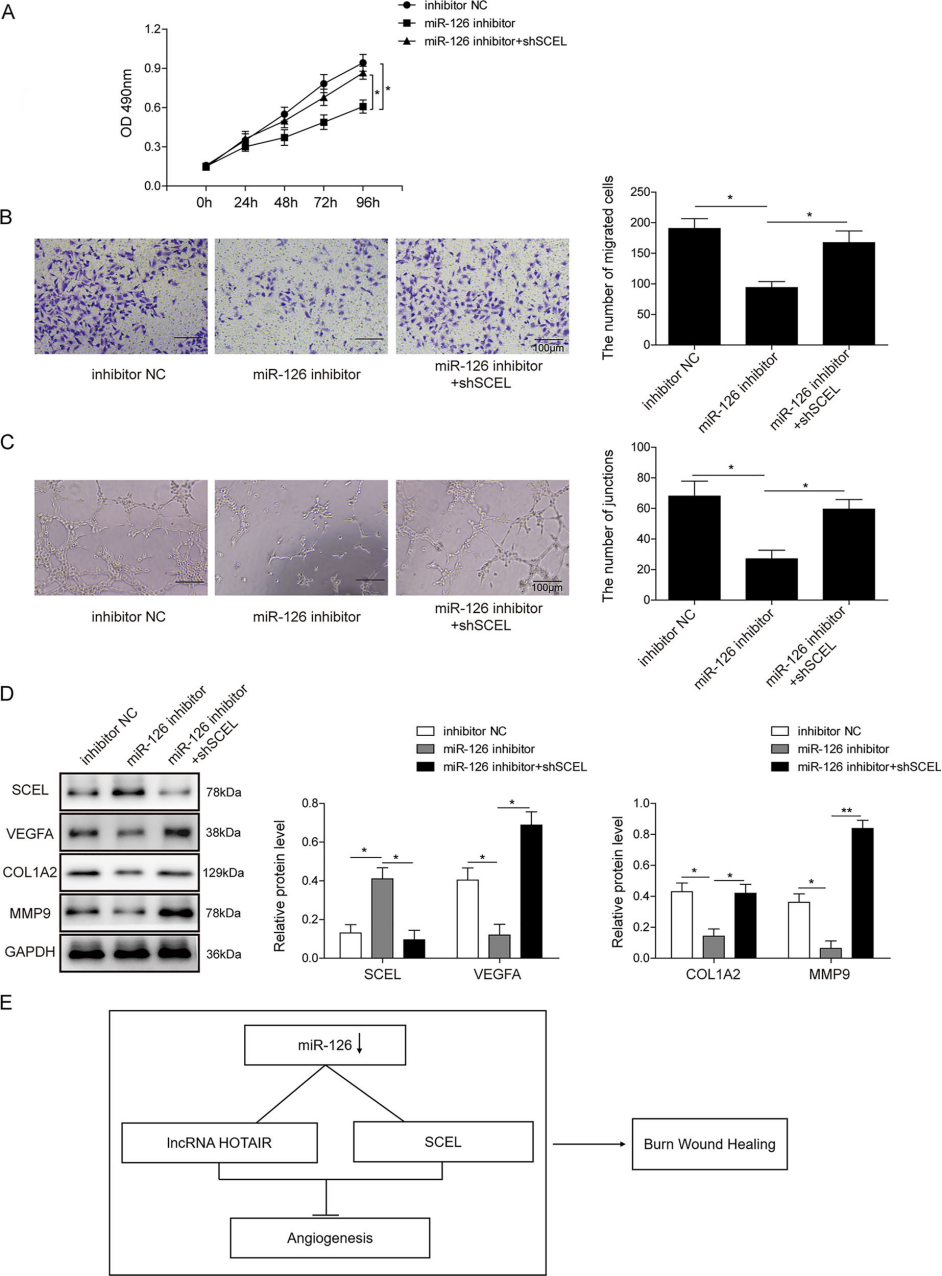
SCEL regulates miR-126-mediated cell proliferation, migration, and angiogenesis in HUVECs. **a** Cell viability was monitored by the MTT assay. **b** Cell migration capacity was assessed by the transwell migration assay. Data were representative images or were expressed as the mean ± SD of *n* = 3 experiments. **c** Angiogenesis was examined by tube formation assays. **d** Western blotting was performed to test the expression levels of SCEL, Col1a2, MMP9, and VEGFA. Data were representative images or were expressed as the mean ± SD of *n* = 3 experiments. **e** A schematic drawing that illustrates the mechanism by which HOTAIR/miR-126/SCEL ceRNA network contributes to burn wound healing, and HOTAIR/miR-126/SCEL axis contributes to burn wound healing through increased angiogenesis. *P < 0.05 and **P < 0.01.

As previous study implied that SCEL is a downstream target of miR-126, the following experiments were conducted to further verify the relationship between miR-126 and SCEL. As shown in Fig. 8a, the capacity of cell proliferation was dramatically repressed by miR-126 inhibitor, while this inhibition was significantly blocked by sh-SCEL transfection. Subsequently, the results of transwell and tube formation assays also presented that silencing of SCEL notably alleviated the suppression of miR-126 inhibitor on cell migration and angiogenesis (Fig. 8b, c), which was further confirmed by western blotting (Fig. 8d). In summary, these data indicated that SCEL might be a downstream effector of miR-126 in burn wound healing.

## Discussion

Millions of patients suffered from severe burn injury worldwide. Slow burn wound healing, infection, pain management, and scar treatment remain as major challenges for burn management and research. Burn wound healing is a complex that involves a number of molecules and pathways. Therefore, it is important to unravel the molecular mechanism(s) of burn wound healing process. Our previous studies have demonstrated that nucleolin promotes proliferation, migration, and angiogenesis of heat-denatured cells^21–23^. We also found that XIST/miR-29a/LIN28A ceRNA network plays crucial roles in denatured dermis repair after thermal injury^24^. In the current study, we identified HOTAIR/miR-126/SCEL ceRNA network, which contribute to burn wound healing. It is worth noting that five pairs of samples with mostly increased miR-126 levels were selected for lncRNA microarray analysis due to the complexity of the bioinformatics analysis. Although HOTAIR/miR-126/SCEL ceRNA network was identified using limited burn-normal tissue pairs, this axis was further validated in the in vitro model, indicating that this regulatory axis is more universal. Future studies are needed to validate this axis in more burn-normal tissue pairs. miR-126 is abundantly expressed in endothelial cells and govern angiogenesis and vascular integrity^7,8^. In endothelial cells, miR-126 is regulated by transcription factors Ets-1 and Ets-2^25^. Knockout of endothelial-specific miR-126 in mice and zebrafish led to leaky blood vessel, hemorrhages, and partial embryonic lethality due to defects in endothelial cell proliferation, migration, and angiogenesis, as well as loss of vascular integrity^8,26^. Consistent with previous knockout studies, we found that miR-126 promoted cell proliferation, migration, tube formation, angiogenesis, and inhibits apoptosis in HUVECs. In recent years, several lncRNAs have been identified as sponges of miR-126 in different cells, such as plasmacytoma variant translocation 1–5 (PVT1–5) and lncRNA activated by transforming growth factor-β (lncRNA-ATB)^27,28^. For instance, lncRNA-ATB has been shown to function as an oncogene by sponging miR-126 in bladder cancer^28^. However, the lncRNA/miRNA/mRNA network in burn wound healing process remains unclear. Here, we demonstrated that miR-126 is highly expressed in burn wound tissues and HUVECs exposed to heat stress. We screened the differentially expressed lncRNAs and mRNAs between normal and burn wound tissues via microarray analysis and focused on the biological function and underlying mechanism of up-regulated miR-126 in burn wound healing process. HOTAIR and SCEL have been identified as a sponge and the direct target of miR-126, respectively.

HOTAIR was originally identified as a cancer-related lncRNA. It is associated with poor prognosis for a variety of cancers, including breast, liver, colorectal, and pancreatic, and so on^29,30^. Aberrant expression of HOTAIR was associated with cancer development, progression, and drug resistance in different cancers^31,32^. A recent study has demonstrated that HOTAIR suppresses placental angiogenesis by inhibiting VEGFA in human placental tissue. In vitro gain-of-function and loss-of-function studies suggest that HOTAIR suppresses cell proliferation, migration, invasion, and tube formation in HUVECs^19^. Our results confirmed these findings by using adenoviral transduction, and we also showed that HOTAIR promoted apoptosis of HUVECs. In addition to the roles of HOTAIR in HUVECs, our functional studies coupled with microarray analysis identified a novel ceRNA network of HOTAIR during burn wound healing process. Collectively, these findings indicate that HOTAIR not only acts as an oncogene but it also plays crucial roles in angiogenesis. In addition, dual luciferase assay coupled with qRT-PCR indicated that HOTAIR directly interacted with miR-126. Functional studies confirmed that HOTAIR served as a sponge of miR-126 in which these two non-coding RNAs exerted opposite effect on cell proliferation, migration, invasion, tube formation, and apoptosis.

SCEL was identified as an arterial intima-enriched gene, and it contributes to vascular adaption to stress^33,34^. Recently, it has been reported that overexpression of SCEL inhibited cell migration and invasion in colorectal cancer cells, and it mediates mesenchymal-to-epithelial transition via activating Wnt signaling^13^. However, the molecular mechanisms by which SCEL regulates angiogenesis remains uninvestigated. In the current study, we demonstrated that overexpression of SCEL inhibits cell proliferation, migration, tube formation, angiogenesis, and promotes apoptosis in HUVECs. To our knowledge, this is the first report regarding the role of SCEL in angiogenesis. Similarly, SCEL has been identified as a direct target of miR-126 by dual luciferase assay and qRT-PCR.

Accumulating evidence has reported the important roles of miRNAs and lncRNAs in wound healing, which add new insights to our understanding of wound complications^35^. Both lncRNAs and miRNAs are potential biomarkers for diagnosis and prognosis of wound healing treatment. For instance, miR-210 serves as a novel molecular marker for chronic wound healing outcomes in a clinical trial^36^. miRNA-based treatment is more effective compared with traditional treatment^37^. With the identification of HOTAIR/miR-126/SCEL axis, future clinical studies could focus on HOTAIR/miR-126-based treatment. These biomarkers might exhibit promising therapeutic effects.

In conclusion, we analyzed a cohort of 20 patients with extensive empyrosis by qRT-PCR. Five pairs of samples with mostly increased miR-126 levels were further subjected to lncRNA and mRNA microarray analysis. Our in vitro data demonstrated that HOTAIR/miR-126/SCEL axis contributes to burn wound healing through increased angiogenesis (Fig. 8e). However, this molecular mechanism needs to be verified in vivo in the future.

## Supplementary information

Supplemental figure legends

Figure S1.

Figure S2

Figure S3

Figure S4.
