# Supplementary material for: Down-regulation of long non-coding RNA HOTAIR promotes angiogenesis via regulating miR-126/SCEL pathways in burn wound healing
Source: Cell Death Dis. 2020 Jan 23;11(1):61. doi: 10.1038/s41419-020-2247-0 (PMC6978466; doi:10.1038/s41419-020-2247-0)
Supplement: Supplementary file 1 — Supplemental figure legends [file 41419_2020_2247_MOESM1_ESM.docx]

**Down-regulation of long noncoding RNA HOTAIR promotes angiogenesis via regulating miR-126/SCEL pathways in burn wound healing**

**Figure S1. Changes in miR-126 levels in HUVECs**. HUVECs were transfected with mimic NC, miR-126 mimics, inhibitor NC or miR-126 inhibitors. miR-126 level was determined by qRT-PCR. U6 served as an internal control. *, P < 0.05.

**Figure S2. Adenoviral transduction efficiency and changes in HOTAIR levels in HUVECs.** HUVECs were transduced with adenovirus. (A) Adenoviral transduction efficiency as measured by GFP. Data were representative images or were expressed as the mean±SD of *n = 3* experiments. (B) HOTAIR level was determined by qRT-PCR. GAPDH served as an internal control. *, P < 0.05, **, P < 0.01.

**Figure S3. Biological function of HOTAIR in HUVECs.** (A) Cell apoptosis was determined by Annexin-V-FITC/PI staining followed by flow cytometry. (B) *In vitro* angiogenesis was quantified by tube formation assays. *, P < 0.05.

**Figure S4. Changes in SCEL levels in HUVECs**. HUVECs were transduced with lentivirus. (A) SCEL level was determined by qRT-PCR. GAPDH served as an internal control. (B) Protein level of SCEL was determined by western blotting. GAPDH served as a loading control. *, P < 0.05, **, P < 0.01.
