# Supplementary figures and images for: Down-regulation of long non-coding RNA HOTAIR promotes angiogenesis via regulating miR-126/SCEL pathways in burn wound healing
Source: Cell Death Dis. 2020 Jan 23;11(1):61. doi: 10.1038/s41419-020-2247-0 (PMC6978466; doi:10.1038/s41419-020-2247-0)

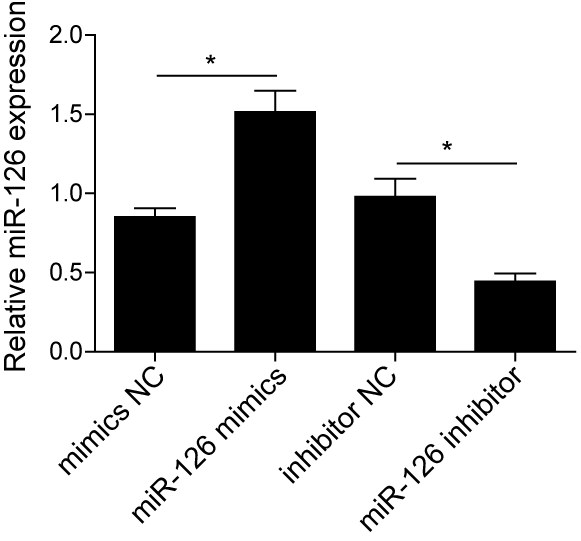

Supplement: Supplementary file 2 — Figure S1. [file 41419_2020_2247_MOESM2_ESM.tif]

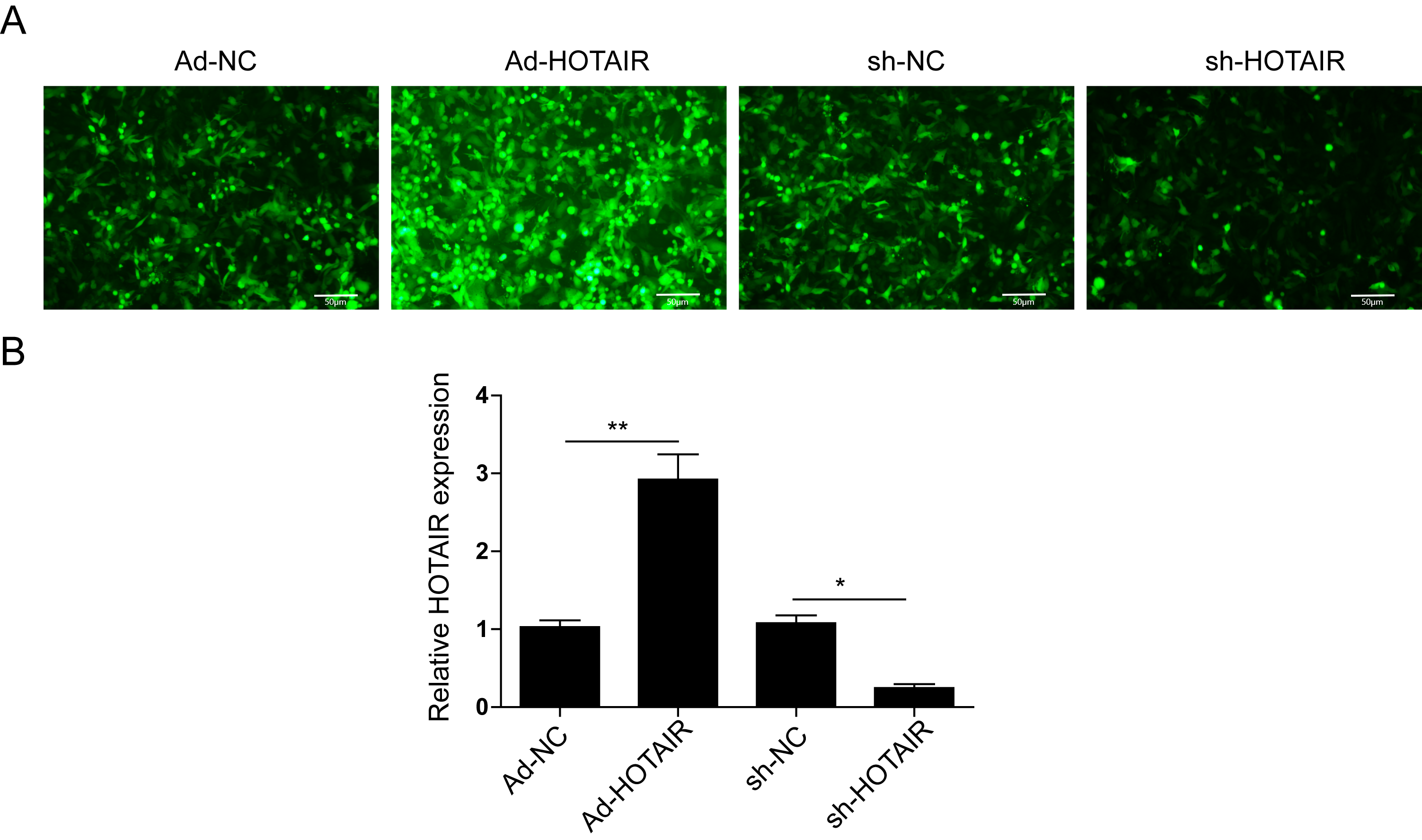

Supplement: Supplementary file 3 — Figure S2 [file 41419_2020_2247_MOESM3_ESM.tif]

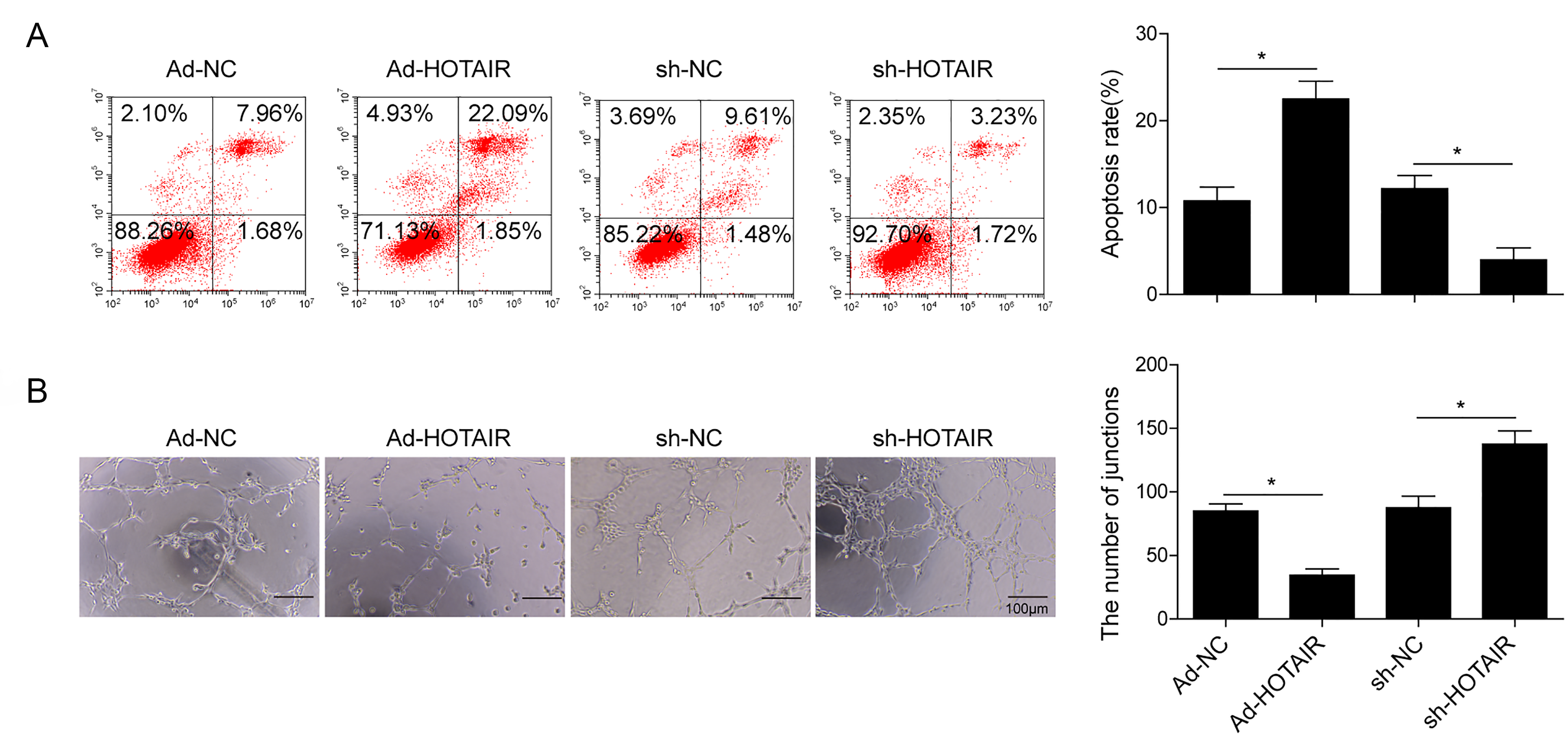

Supplement: Supplementary file 4 — Figure S3 [file 41419_2020_2247_MOESM4_ESM.tif]

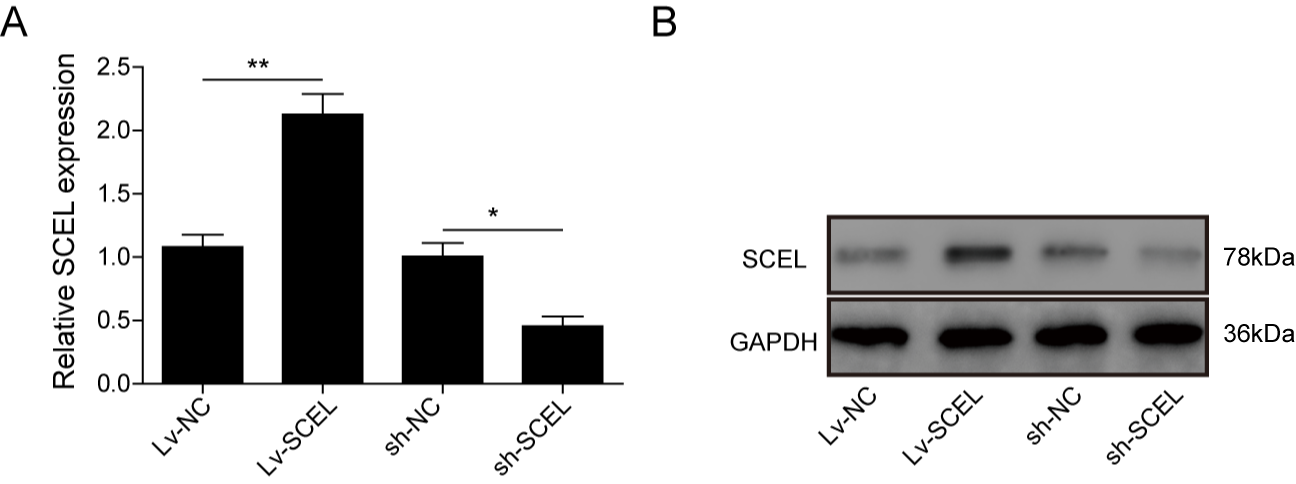

Supplement: Supplementary file 5 — Figure S4. [file 41419_2020_2247_MOESM5_ESM.tif]
